# Using CNN Saliency Maps and EEG Modulation Spectra for Improved and More Interpretable Machine Learning-Based Alzheimer's Disease Diagnosis

**DOI:** 10.1155/2023/3198066

**Published:** 2023-02-08

**Authors:** Marilia Lopes, Raymundo Cassani, Tiago H. Falk

**Affiliations:** ^1^Institute National de la Recherche Scientifique (INRS-EMT), University of Quebec, Montreal, Canada; ^2^McConnell Brain Imaging Centre, Montreal Neurological Institute, McGill University, Montreal, Canada

## Abstract

Biomarkers based on resting-state electroencephalography (EEG) signals have emerged as a promising tool in the study of Alzheimer's disease (AD). Recently, a state-of-the-art biomarker was found based on visual inspection of power modulation spectrograms where three “patches” or regions from the modulation spectrogram were proposed and used for AD diagnostics. Here, we propose the use of deep neural networks, in particular convolutional neural networks (CNNs) combined with saliency maps, trained on power modulation spectrogram inputs to find optimal patches in a data-driven manner. Experiments are conducted on EEG data collected from fifty-four participants, including 20 healthy controls, 19 patients with mild AD, and 15 moderate-to-severe AD patients. Five classification tasks are explored, including the three-class problem, early-stage detection (control vs. mild-AD), and severity level detection (mild vs. moderate-to-severe). Experimental results show the proposed biomarkers outperform the state-of-the-art benchmark across all five tasks, as well as finding complementary modulation spectrogram regions not previously seen via visual inspection. Lastly, experiments are conducted on the proposed biomarkers to test their sensitivity to age, as this is a known confound in AD characterization. Across all five tasks, none of the proposed biomarkers showed a significant relationship with age, thus further highlighting their usefulness for automated AD diagnostics.

## 1. Introduction

Alzheimer's disease (AD) is a degenerative brain disease and the most common cause of dementia [1, 2]. AD progression leads to the loss of cognitive (e.g., memory, reasoning, and communication) and behavioral functions that will ultimately interfere with the individual's daily life. Diagnosing AD can be challenging, as the disease can initiate pathophysiological processes 20 years before any clinical symptoms appear [3, 4]. As such, improving early diagnostics has become a fundamental element in AD research and therapy [5], especially since there are currently no cures for AD [6–8]. While a definite diagnosis for AD can only be achieved through a postmortem structural examination of the brain, clinical diagnosis currently relies on the use medical history and exams, such as the mini-mental state examination (MMSE) [9] and the clinical dementia rating (CDR) [10]. Moreover, a handful of biomarkers have also been incorporated into the clinical diagnostic process, including structural neuroimaging (e.g., magnetic resonance imaging (MRI)-based measures of hippocampal volumes) [11, 12], blood and urine samples [13], cerebral spinal fluids (e.g., tau and beta-amyloid levels) [14], and genetic risk profiling [15]. More recently, there has also been a push to incorporate biomarkers extracted from electroencephalograms (EEG) into the diagnostic process [16].

Indeed, EEG has proven to be a useful tool in the study of AD, with several advantages over other neuroimaging modalities, including noninvasiveness, lower cost, the possibility to detect early synaptic dysfunction prior to symptoms arising, and the possibility to more easily track the course of the disease [17]. EEGs record from the scalp the electrical field produced by the interaction of neurons. Most of the published works have relied on the analysis of resting-state EEG (rsEEG) in either eyes-open or eyes-closed conditions, thus making the data collection procedure more comfortable for elderly patients [18]. Over the years, several changes in the EEG have been reported as a function of AD, namely, slowing of the EEG, reduction in signal complexity, perturbations in interelectrode synchrony/coherence, and a neuromodulatory deficit [19], resulting from the reduction of neurotransmitters due to damage to neuro-pathways by the disease. More recently, machine learning tools applied to conventional EEG spectral features have started to show promising results for AD diagnosis (e.g., see [20–22]); in some cases, accuracy around 90% has been reported.

In regards to features, typically rsEEG-based AD studies extract diagnostic features from the five traditional EEG frequency subbands, namely, delta (*δ*=0.1–4Hz), theta (*θ*=4–8Hz), alpha (*α*=8–12Hz), beta (*β*=12–30Hz), and gamma (*γ* > 30Hz). The work in references [23, 24], in turn, proposed the measurement of the spectrotemporal dynamics of each of these subbands and showed that improved diagnostic accuracy could be achieved. The resultant 2-dimensional (frequency vs. frequency) power modulation spectrogram was shown to not only improve accuracy but to also be more robust to artifacts commonly observed with EEGs [25]. The use of these conventional frequency bands, however, may not be optimal for AD analysis, as they have been defined based on visual inspection of EEG signals in healthy subjects [26]. In fact, the work in references [27, 28] showed that nontraditional bands were better for AD diagnostics. Building on these insights, the work in reference [29] used visual inspection to find optimal power modulation spectrogram regions or “patches” that improved diagnostic accuracy relative to conventional measures widely used in AD characterization.

In this article, we build on the work of [29] and propose the use of a data-driven method in order to find the optimal modulation spectrogram patches for AD diagnostics, whereas in [29], visual inspection was used. Visual inspection can lead to the loss of discriminatory information and potential biases, as well as reduce the interpretation of the results. In particular, we propose the use of saliency maps obtained from a convolutional neural network (CNN) trained to detect AD. Saliency maps provide insights into which regions of the input image the CNN is focusing on to make its decisions [30] and could improve the interpretability of the obtained results. While the use of saliency maps has been explored for AD classification based on MRIs [31, 32], it has yet to be explored for EEG data. Given the modulation spectrogram 2-dimensional representation and its improved discrimination relative to the conventional spectrogram, it can be a prime candidate for a saliency map-based biomarker. Experiments described herein show the usefulness of the proposed method not only in detecting AD but also in predicting AD severity level. Finally, the measure is shown to not be affected by participant age, a common confounding factor in AD studies.

The remainder of this paper is organized as follows: Section 2 describes the materials and methods used in our experiments. Section 3 presents the experimental results and discusses them in light of the existing literature, including study limitations. Lastly, conclusions are presented in Section.

## 2. Materials and Methods

### 2.1. Participants

We rely on EEG data collected from fifty-four participants recruited from the Behavioral and Cognitive Neurology Unit of the Department of Neurology and the Reference Center for Cognitive Disorders at the Hospital das Clinicas in São Paulo, Brazil. AD diagnosis and identification of severity level (mild-AD or moderate-to-severe AD) were performed by experienced neurologists according to NINCDS-ADRDA criteria [33] and classified based on the Brazilian version of the MMSE [34]. Ethics approval was obtained from the Research Ethics Office, and all participants consented to participate in the study. The data have been divided into three groups: (i) “*N*” consists of 20 healthy elderly controls, (ii) “AD1” corresponds to 19 mild-AD patients, and (iii) “AD2” to the 15 patients diagnosed with moderate-to-severe AD. In experiments where all AD patients are combined (i.e., AD1 + AD2), this group will be termed “AD”.  Table 1 shows participant demographic details, including MMSE scores.

Inclusion criteria for the *N* group included MMSE score ≥ 25 and CDR score = 0, as well as no indication of functional cognitive decline. The AD1 group inclusion criteria, in turn, included MMSE ≤ 24 and 0.5 ≤ CDR ≤ 1; whereas for the AD2 group, they included an MMSE ≤ 20 and CDR score = 2. An additional criterion used for the two AD groups was the presence of functional and cognitive decline over the previous 12 months based on detailed interviews with knowledgeable informants. Exclusion criteria included diabetes mellitus, kidney disease, thyroid disease, alcoholism, liver disease, lung disease, or vitamin B12 deficiency, as these conditions can also cause cognitive decline.

### 2.2. EEG Acquisition and Preprocessing

For the acquisition of EEG signals, twenty channels were used following the 10–20 international mounting system. EEG signals were recorded with 12 bit resolution and 200 Hz sampling frequency using BrainTech 3.0 instrumentation (EMSA Equipamentos Médicos INC., Brazil). In addition, electrode impedance was kept below 10 *k*Ω and attached bi-auricular (A1 and A2) electrodes were used as reference. A resting-state eyes-closed (rsEEG) protocol was followed, and data were recorded for at least eight minutes for each participant.

Based on insights from reference [25], raw EEG was preprocessed with a zero-phase FIR bandpass filter with a bandwidth 0.5–45 Hz, followed by processing with the wavelet-enhanced independent components analysis (wICA) step to remove eye movement and/or muscle artifacts [35, 36].

### 2.3. Power Modulation Spectrogram

**Figure 1:**
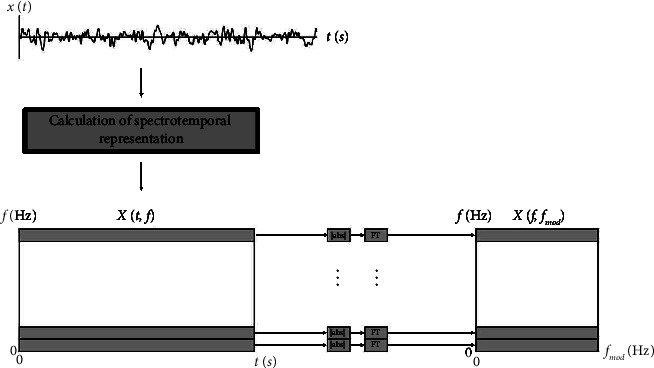
Signal processing steps for the computation of the EEG power modulation spectrogram. (Image adapted from [29]).

The 2-dimensional power modulation spectrogram representation has been proposed to characterize spectrotemporal changes in the EEG due to AD (e.g., see [37]). The signal processing steps involved in the computation of the representation can be seen in Figure 1. First, the EEG time series *x*(*t*) is processed by a time-frequency mapping to generate the time-frequency representation *X*(*t*, *f*). This mapping can be a short-time Fourier transform, for example, or a wavelet transform. In order to measure the temporal dynamics of each spectral bin, a second transformation is done on the time-frequency signal, now across the time dimension. This is achieved with a Fourier transform (FT) of the instantaneous power of each spectral bin, resulting in the power modulation spectrogram *X*(*f*, *f*_mod_), i.e.,

(1) $$\begin{array}{c} X\left(f,f_{\mathrm{mod}}\right)=F_{t}\left\{{\left|X\left(t,f\right)\right|}^{2}\right\}\text{,} \end{array}$$

where *ℱ*_*t*_{·} indicates the Fourier transform over the time dimension and *f*_mod_ indicates the modulation frequency dimension. The final result is a frequency-modulation frequency spectral representation that describes the second-order periodicities that would not be present in conventional spectral or time-frequency representations [38, 39].

**Figure 2:**
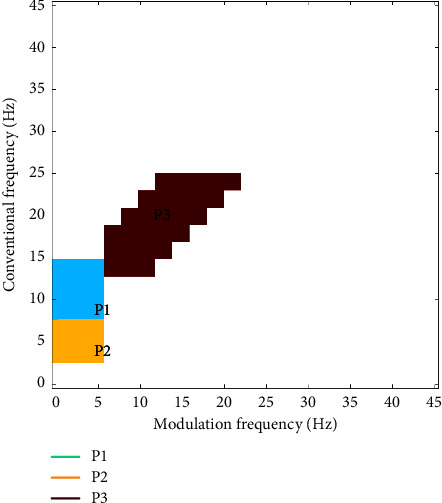
Three modulation spectrogram patches proposed in [29].

In its original version, frequency bins in this 2-dimensional representation were grouped across the conventional and modulation frequency axes into the five traditional subbands, namely, delta, theta, alpha, beta, and gamma, and each frequency-modulation frequency bin was used as a feature for AD detection (e.g., see [23, 37]). More recently, the work in reference [29] showed that the use of the traditional bands was not optimal for the task at hand, and through visual inspection, three new regions were defined and proposed, termed “patches,” as shown in Figure 2. The modulation energy computed from these three patches, as well as their ratios, was proposed as new features for AD diagnosis and severity level prediction. Experimental results showed their superiority to the traditional band-grouping strategy, but suggested that some of the patches could be a result of normal aging. In this work, we build on these patches and propose a data-driven manner to explore if optimal patches can be found that are indicative of AD and not normal aging. Experiments rely on a 45 × 45 power modulation spectrogram, where each bin corresponds to 1 Hz resolution.

### 2.4. Convolutional Neural Networks

Deep learning has emerged as a very powerful pattern analysis tool over the last decade. Increases in computational power have allowed for artificial deep neural networks (DNNs) with multiple hidden layers and billions of parameters to be trained within reasonable time frames. This increase in computational capacity has resulted in the emergence of a number of new deep neural network architectures, such as convolutional neural networks (CNNs), recurrent neural networks (RNNs), and recursive neural networks (RvNNs), to name a few [40]. Here, we rely on CNNs and saliency maps to extract new biomarkers of AD from EEG modulation spectrograms; hence, a brief description of CNNs is given for the sake of completeness; more details can be found in [41–44].

While feed-forward DNNs multiply the inputs by optimized weights obtained during the training, CNNs include layers that perform convolutions, i.e., the dot product of the convolution kernel with the layer's input matrix. The convolution kernel slides along the input matrix for the layer, thus generating a new feature map that contributes to the input of the subsequent layer. This is followed by other layers such as pooling layers, fully-connected layers, and normalization layers [45]. CNNs have resulted in state-of-the-art image recognition performance [46], as each convolution layer extracts specific features from the image, such as vertical and horizontal lines, color and shape, contrast, exposure variations, or image borders, to name a few. Using sequential convolution layers, each new feature map builds on the properties captured by the previous map. Depending on the number of layers used and the type of data used during training, CNNs may even learn features that take care of preprocessing, detection, and recognition, thus enabling end-to-end systems. When used for biomarker development, their use may also result in regions that could be more robust to EEG artifacts.

While many deep learning models are regarded as “black boxes,” providing limited insights on what parts of the input image are being used for discrimination, so-called saliency maps have been proposed to overcome this limitation. This method measures the spatial support of a particular class in each image via a heatmap. Saliency maps have been used for region-of-interest extraction [47], medical imaging [48, 49], robot vision [50], and audio-visual integration [51, 52], in addition to AD diagnosis based on MRI [32]. Saliency maps are obtained by computing the gradient of the output category in relation to the input image. In this way, the impact of how the value of the output category changes in relation to a small change in the pixels of the input image is observed. The highlighted regions in the resulting map indicate that they are important areas for the classification provided, where a small change in that pixel would change the classification relative to other pixels. In essence, saliency maps are constructed by back-projecting the information corresponding to the identified class, thus allowing us to visualize image regions that mostly affect prediction. Here, the keras-vis [53] toolkit was used to extract saliency maps.

It is hypothesized that using saliency maps with EEG power modulation spectrograms will allow for new biomarkers of AD to be developed in a data-driven manner and for comparisons with visually inspected regions to be made. Unlike simple images, however, EEGs are extracted across multiple channels, each generating its own image-like modulation spectrogram. As information from multiple channels is taken into account during classification, 20 different saliency maps, one for each EEG channel can be generated. While each of these maps could be used separately, here, for simplicity, we take their overall average and use the aggregated map for analysis; use of individual channel maps is left for a future study. In our experiments, a CNN with two convolution layers was used, having as input a tensor of 45 × 45 × 20 dimensionality. The kernel size is equal to 3 × 3 and a dropout rate of 85% was used. ReLU activation functions were used for the convolution layers. In the fully-connected layers, in turn, LeakyReLU was used as an activation function. A total of three fully-connected layers were used, including the last classification layer. Hyper-parameter tuning was performed, and Table 2 presents the tested parameters and final values used.

### 2.5. Proposed Method

**Figure 3:**
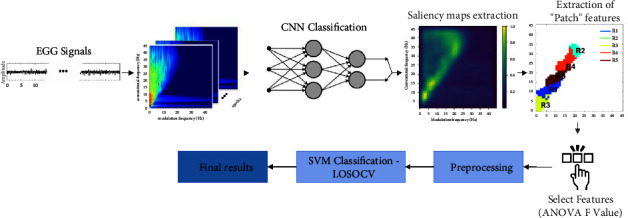
Overview of the proposed system architecture.

Figure 3 depicts the block diagram of the proposed method. First, EEG signals are segmented and transformed to the power modulation spectrogram domain which is then z-normalized. Segments of 8-second duration with 1-second overlap are taken; a minimum of 460 segments are available per subject. CNNs are then trained on five different classification tasks, namely, Task 1 (T1): *N* vs. AD1 vs. AD2 (multi-class discrimination); Task 2 (T2): *N* vs. AD (AD diagnosis); Task 3 (T3): *N* vs. AD1 (early AD detection); Task 4 (T4): AD1 vs. AD2 (AD progression); and Task 5 (T5): *N* vs. AD2 (late-stage AD detection). Saliency maps are then extracted for each of the five tasks to indicate regions of importance for each task. To allow for comparisons with the three visually obtained regions [29], a clustering algorithm is applied on the saliency map “islands” to obtain new optimal patches. These patches are then used for classification. In the subsections to follow, more details about the clustering method and final classification steps are given. Algorithm 1 shows an overview of the processing steps involved in the feature extraction and train/testing stages of the proposed method.

### 2.6. Saliency Map Clustering

After the CNNs are trained, saliency maps are extracted from the last dense layer. Saliency maps from each training input are obtained and averaged over all training samples to obtain one final map. Here, we are interested in using the maps to find optimal regions in the modulation spectrogram for new biomarker development. To this end, we use thresholding and clustering to find the optimal number of patches, in a data-driven manner, for the particular task at hand. To find the optimal clusters per task for this final map, we propose to first take the difference between the average modulation spectrograms of the two groups in a given classification task and use the saliency map as a mask to be applied to this differential spectrogram. When clustering, two parameters are explored via grid search, namely, the saliency value threshold and the number of clusters. Threshold values from 80–96% were explored, with a hop of 2% and 3–5 clusters were tested. We selected three as the minimum to coincide with the patches proposed in [54] and five to strike a balance with feature dimensionality. Clustering was performed via the k-means algorithm in MATLAB.

Four distinct masking approaches are tested to explore their impact on overall accuracy. They vary based on which task the saliency map was obtained from, e.g., more generic, as in Task 2, to more specific, as in Task 3. These four different experiments are detailed as follows:

- Experiment 1: This experiment takes the generic *N* vs. AD task and uses the salience map obtained from the three-class task *N* vs. AD1 vs. AD2 as a mask.
- Experiment 2: This second experiment is a bit more tuned to the task at hand as it considers individual subclasses directly, while still using the most generic saliency map. In particular, the difference signal is taken for each binary task, namely, *N* vs. AD1, N vs. AD2, and AD1 vs. AD2. The same saliency map as Experiment 1 is used.
- Experiment 3: This is the most specialized of the experiments as the saliency maps corresponding to each differential modulation spectrograms are used to find the optimal clusters. For example, saliency maps found for the *N* vs. AD1, *N* vs. AD2, and AD1 vs. AD2 tasks are used with differential spectrograms obtained from *N* vs. AD1, *N* vs. AD2, and AD1 vs. AD2 classes, respectively.
- Experiment 4: This experiment builds on Experiment 1 and takes the *N* vs. AD task and uses the salience map obtained from the same *N* vs. AD mask, which can be seen as the most generic.

### 2.7. Biomarker Selection and AD Diagnosis

Once the optimal number of clusters is found, these regions will become candidate patches for each of the five tasks. As in [29], the modulation spectrum power *R*_*i*_ in patch/cluster *i* is computed as follows:

(2) $$\begin{array}{c} R_{i}=\iint_{RG_{i}}={\left|X\left(f,f_{\mathrm{mod}}\right)\right|}^{2}df\;df_{\mathrm{mod}}\text{,} \end{array}$$

where *RG*_*i*_ corresponds to the new patch found by cluster *i*. As in [54], the power ratios between different patches are also treated as features and these are computed across all 20 channels.

As can be seen, the number of candidate features/biomarkers can quickly grow with an increasing number of channels and regions, causing potential curse-of-dimensionality issues with the downstream classification tasks. As such, feature selection is needed to reduce the final number of features to a reasonable number. Previous EEG-based works have relied on 24 input features (e.g., see [23, 25]), and we follow this procedure to allow for fair comparisons with prior works. We use 25% of the training set (described in the next subsection) to find the best 24 features using an ANOVA *F*-value metric computed between each feature and class label.

Previous works have relied on a support vector machine (SVM) classifier to map patch features to a final diagnostic decision. For consistency, we also use an SVM for final AD classification, thus allowing for a more fair comparison with previous work. This also assures that the performance gains achieved are a result of the improved biomarkers and not of an improved classifier (e.g., a CNN itself). SVMs use kernels to map data from two classes into a higher dimension in which a hyperplane can be used to separate the two different classes by a certain margin [55]. Different kernels provide different properties and allow for more complex class partitions to be found. Here, a radial basis function (RBF) kernel with gamma *γ* = 1/24, where 24 is the number of features, and *C* = 1 are used as hyper-parameters and tuning is not performed to gauge the benefits of the features themselves, and not on the classifier. Prior to classification, the top-24 new biomarkers are normalized between [−1, 1]. Final testing follows a leave-one-subject-out (LOSO) plus bootstrapping procedure using the disjoint test set described below. With the LOSO-plus-bootstrapping setup, for each subject, the classifier is trained with data from *N* − 1 subjects that are randomly sampled and repeated ten times.

### 2.8. Testing Setup

**Figure 4:**
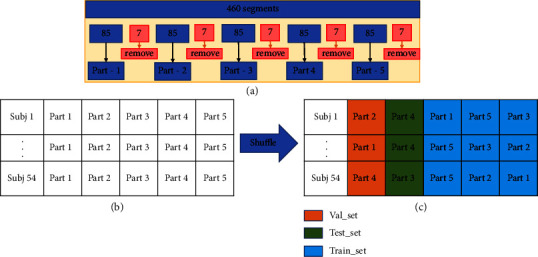
Steps to perform database partitioning to avoid information leakage. (a) First, 460 segments per subject are split into 5 parts of 92 segments. The last seven are discarded to avoid data leakage. (b) Disjoint parts are then combined per subject. (c) Lastly, temporal shuffle is done to avoid ordering effects. 1/5, 1/5, and 3/5 of the shuffled data are partitioned into validation, testing, and training subsets.

The available dataset has to be split to allow for CNN training for biomarker selection as well as for SVM training for final classification. As such, the available data needs to be partitioned in such a way that data leakage does not occur into the final test set. To this end, the data partitioning scheme illustrated in Figure 4 is performed to avoid any data leakage into the disjoint training, validation, and test sets. First, the 460 segments are partitioned into five parts, each with 92 segments. Since the segments are obtained with a 7-second overlap, the last 7 segments of each part are discarded, to avoid information leakage between folds (see Figure 4(a)). Each of the five parts is comprised of 85 segments, for each of the 54 subjects (Figure 4(b)). Finally, a time-dimension shuffle was made to avoid any ordering effects on the data. From the shuffled data, 1/5 of the data was set aside for validation, 1/5 for testing, and 3/5 for training (Figure 4(c)).

### 2.9. Figures-of-Merit and Benchmark Method

We use accuracy and *F*1-score as figures-of-merit to gauge the performance of the proposed method and compare against the visual-inspection-based biomarkers from [29]. Accuracy represents the ratio of correct predictions to total predictions, i.e.,

(3) $$\begin{array}{c} Accuracy=\frac{TP+TN}{TP+TN+FP+FN}\text{,} \end{array}$$

where TP stands for true positive (target label is correctly predicted), TN for true negative (nontarget label predicted correctly as nontarget), FP for false positive (nontarget label predicted as target), and FN for false negative (target label erroneously predicted as nontarget).

In turn, *F*1-score is given by the weighted average between recall and precision, namely,

(4) $$\begin{array}{c} F1=\frac{2*precision*recall}{precision+recall}\text{.} \end{array}$$

*F*1-scores are useful for unbalanced datasets, such as the one used here. For completeness, precision identifies how accurately the model predicted the positive classes, i.e.,

(5) $$\begin{array}{c} Precision=\frac{TP}{TP+FP}\text{,} \end{array}$$

where recall measures the ratio of the number of true positive events to the sum of true positive and false negative events, i.e.,

(6) $$\begin{array}{c} Recall=\frac{TP}{TP+FN}\text{.} \end{array}$$

As for the benchmark, we use the state-of-the-art system described in [29] for comparisons.

### 2.10. Age-Related Confounds

Age is a known confounding factor and risk factor for AD [56, 57]. Since the healthy and patient populations in our dataset are not age-matched, we need to be careful not to propose features that are correlates of normal neural deterioration due to aging (e.g., see [58, 59]), but instead, related to neurodegeneration due to the disease. First, we explore how well the top-24 features can be used to predict age via a linear regression. To test if the top-selected features carry age-related information, we compare them against a random age prediction regressor. With this random linear regressor, we shuffle the age of participants in a random manner. For this task, we combine all the three dataset partitions and split them into train/test using 75% and 25% of the data, respectively. This partition is run 100 times, and the average root mean square error (between true and predicted age) and standard deviations are reported. To assure that the differences with the random regressor are not significant, a *t*-test is performed with a 99% confidence level (*p* > 0.01); the Python statistical package sciPy.stats was used for this test.

## 3. Results and Discussion

### 3.1. CNN Accuracy

First, we explore how well the CNN is behaving on the validation set in order to gauge the effectiveness of the obtained saliency maps for downstream AD detection on the unseen test set.  Table 3 reports the results obtained for each of the five classification tasks. We use only accuracy (Acc) and the *F*1-score for this analysis. Overall, the *N* vs. AD2 (T5) task resulted in the highest accuracy and *F*1 score. This is expected, as the neural changes with moderate-to-severe AD are likely to be the greatest compared to healthy EEG, thus making it easier for the CNN to distinguish them. The accuracies obtained are in line with those reported previously for manually-selected clean EEG segments (e.g., see [23]), hence providing confidence on the potential of the CNN to find discriminatory features for AD classification, thus validating the use of the salience maps for feature extraction and potentially artifact rejection.

### 3.2. Saliency Maps

**Figure 5:**
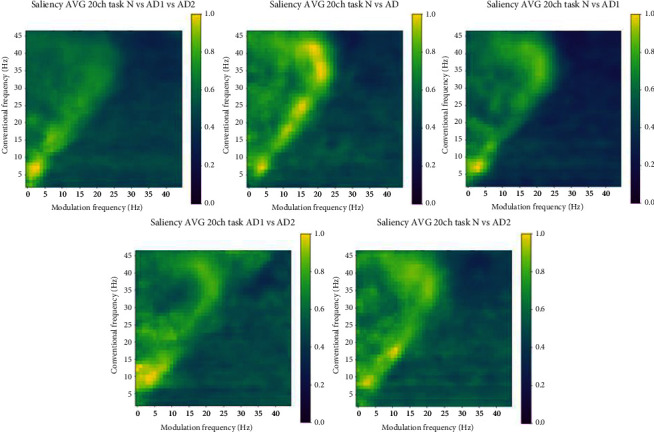
Average saliency map for each of the five tasks.

With the CNN approach validated, we proceed to investigate the saliency maps generated from the EEG channels. While each individual channel map could be used for channel-specific feature generation, here, for simplicity, we explore the use of only one general mask for all channels. As such, the average map is used.  Figure 5 depicts the average map found for each of the five tasks. At a first glance, similar regions to the patches reported in reference [29] (shown in Figure 2) are seen, but with additional regions also showing importance. Next, we explore the best threshold and number of clusters for each of the four experiments tested.

### 3.3. Saliency Map Clustering for Patch Detection

**Figure 6:**
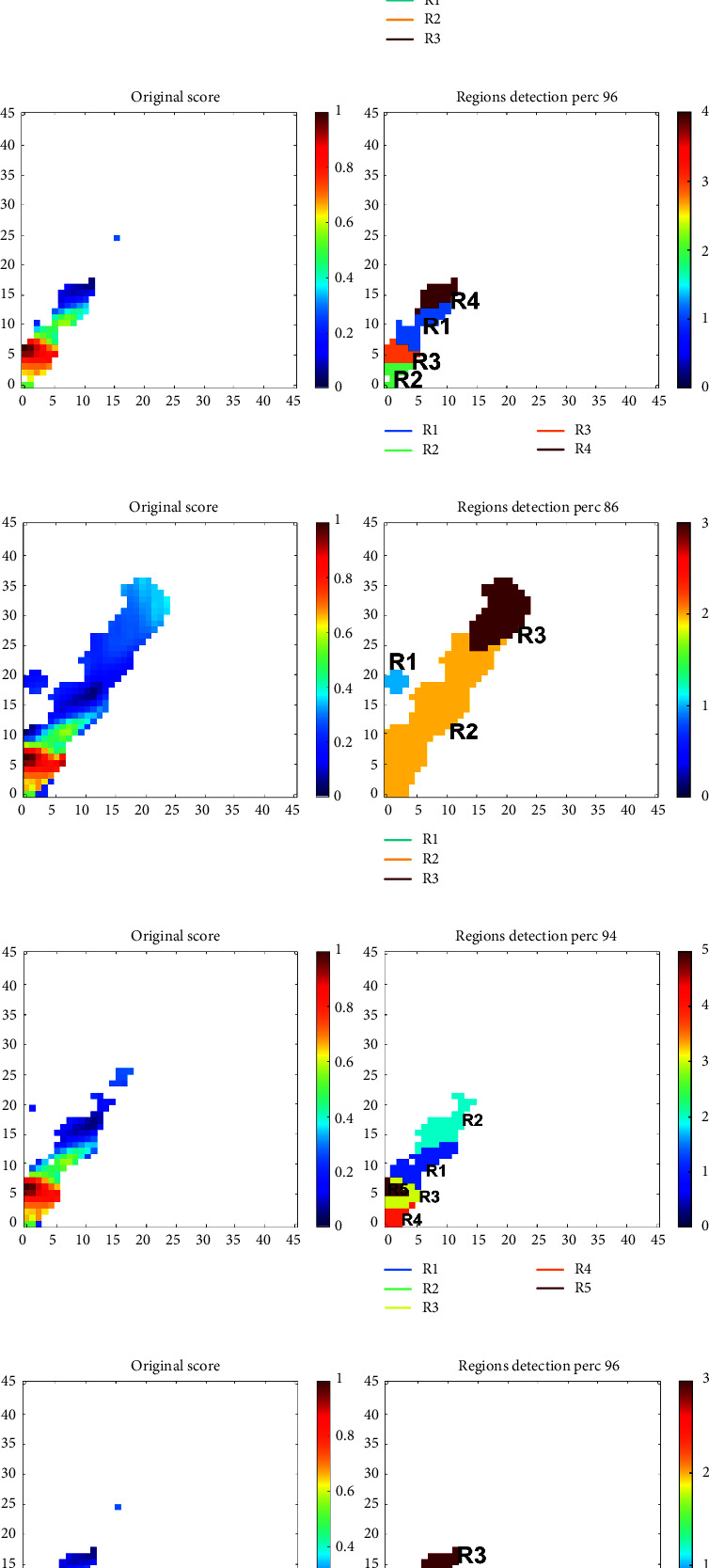
Optimal regions and clusters found for each of the five tasks using experiment 1 settings. In each subplot, the *y*-axis corresponds to conventional frequency (unit: Hz) and the *x*-axis to modulation frequency (unit: Hz). (a) *N* vs. AD1 vs. AD2, (b) *N* vs. AD, (c) *N* vs. AD1, (d) AD1 vs. AD2, and (e) *N* vs. AD2.

**Figure 7:**
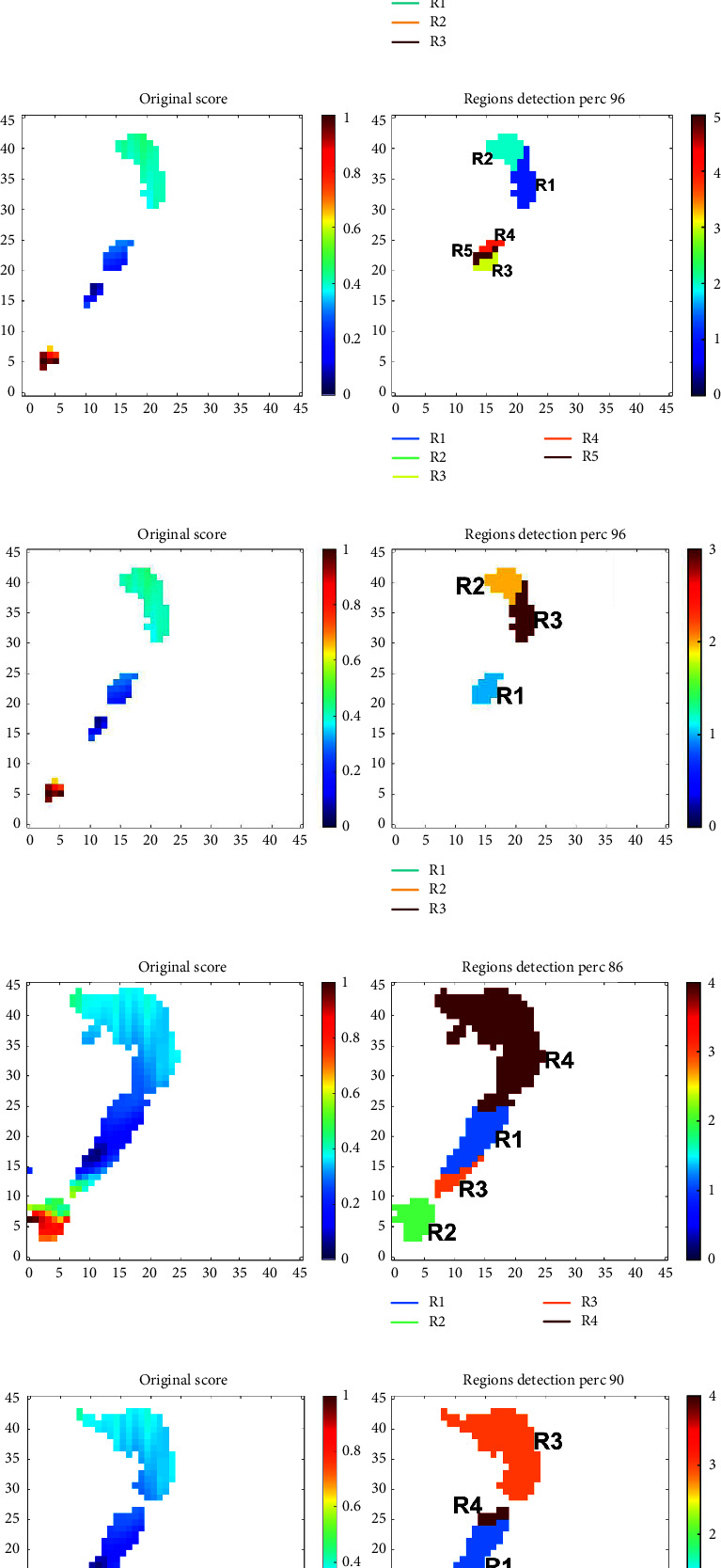
Optimal regions and clusters found for each of the five tasks on the experiment 4 settings. In each subplot, the *y*-axis corresponds to conventional frequency (unit: Hz) and the *x*-axis to modulation frequency (unit: Hz). (a) *N* vs. AD1 vs. AD2, (b) *N* vs. AD¸ (c) *N* vs. AD1, (d) AD1 vs. AD2¸ and (e) *N* vs. AD2.

Table 4 shows the best thresholds (Th) and number of clusters (C) found for each of the four experiments; henceforth, these combinations are used. The optimal patch regions found based on these parameters can be seen in Figures 6 and 7 for Experiment 1 and Experiment 4, respectively. The plots of the other two experiments are omitted for brevity, but similar regions were found. In each subfigure of these plots, the *x*-axis corresponds to modulation frequency, the *y*-axis to conventional frequency, the left-most plot shows the patches found with the optimal threshold, and the right-most plot shows the optimal clusters found. In each image, the found clusters/regions for each task are labelled as “*R*_*i*_.” It is important to emphasize that each task achieved different regions of importance, hence, e.g., *R*_1_ from Task 1 may differ from *R*_1_ from Task 2. As such, when listing the rank of top features in tables to follow, we use a subscript from 1–5 to remind the reader that the region listed is related to its corresponding counterpart seen in Figures 6 and 7.

As can be seen, the patches found using Experiment 1 settings resemble closer those found via visual inspection in reference [29], especially P2 and P3 seen in Figure 2. Other than the *N* vs. AD1 task, the obtained regions look fairly alike for all tasks. One region not used before but that was shown to be important in this data-driven analysis method corresponds to patches in lower frequencies, below 5 Hz (delta and low theta), corroborating some earlier findings from reference [23]. Experiment 4 settings, in turn, showed similar regions to those obtained from visual inspection but also included across all tasks important information extracted from higher frequencies (around the beta and gamma frequency ranges), a finding not seen previously in modulation spectral studies.

### 3.4. Feature Importance

The optimal patches for each classification task have now been found and they serve as masks for biomarker extraction from each EEG channel. To reduce the number of features being used by the SVM classifier, feature ranking is performed and only the top-24 features are kept.  Table 5 lists the top features for each of the five tasks for the Experiment 1 setting. The notation RXoRY is used to indicate the feature corresponding to the ratio of the modulation power in patch RX to the modulation power in patch RY. Subscripts 1–5 indicate that the regions are those corresponding to Tasks 1–5. It can also be seen that for the majority of the tasks, frontal region electrodes stood out. Interestingly, as reported in reference [25], frontal regions are usually discarded with EEG analysis relying on visual inspection of EEGs, as these areas are often contaminated by eye movement artifacts. Our findings suggest that such important regions may be again incorporated into EEG analysis by utilizing automated artifact removal algorithms, such as wICA, and features that could be inherently more robust to artifacts. For the AD1 vs. AD2 task, most features belong to the frontal and central electrodes, a region known to be affected by the progression of AD [60], likely due to the expansion of the atrophy into the superior parietal and frontal cortex [61]. For the *N* vs. AD and *N* vs. AD1 tasks, in turn, features extracted from the temporal brain region stood out, corroborating studies showing atrophy in cortical regions in the temporal regions [62]. In particular, in the *N* vs. AD1 task, temporal and frontal brain regions stood out; such fronto-temporal regions have been used for early AD diagnosis in the past [63].

As for the Experiment 4 setting, Table 6 shows the top-24 features selected. Unlike the Experiment 1 settings, high-frequency range features (both in conventional and modulation frequency domains) appear in the top-5 features for three of the five tasks, namely, *N* vs. AD, *N* vs. AD1, and AD1 vs. AD2. This suggests that such frequency ranges could be useful for early diagnosis and AD progression assessment, hence, providing new insights not previously achieved via visual inspection. Some research has shown the importance of gamma band activity in AD [64], though this region is often corrupted by muscle artifacts. The findings obtained here suggest that the temporal dynamics of the beta and gamma bands may provide some discriminatory information and that a more generic mask may capture the dynamics of a band that is typically discarded due to artifacts, hence further emphasizing the robustness of the new biomarkers. In addition, some of the regions found belong to the beta-m-alpha and gamma-m-alpha frequencies. These frequencies have been shown in [65] to correlate with cerebral hemodynamics information conveyed by functional near-infrared spectroscopy in regions related to impaired blood flow in AD [66, 67], thus providing some additional interpretability to the selected biomarkers.

With these settings, it can be seen that most of the features were derived from electrodes over the temporal, parietal, and occipital regions, thus in agreement with [62]. An occipital brain region was most important for two of the five tasks (*N* vs. AD and *N* vs. AD1) and was the second most important region in the AD1 vs. AD2 task, hence corroborating classical findings from reference [68]. Unlike these classical studies, however, where occipital changes were found mostly in lower frequency ranges, here we observe them to be extracted from higher ranges around beta conventional frequency and gamma modulation frequencies, hence a finding also not previously found via visual inspection. For space limitations, we omit the features selected via the other two experiments, but they were similar to those found in Experiment 1 and 4.

### 3.5. Overall Classification Accuracy

Lastly, using the top-24 features, Table 7 shows the final accuracy and F1 scores obtained for each of the four experiments and five classification tasks. The results achieved with the benchmark, i.e., the visually obtained power modulation spectrogram patch features from reference [29], are also added for comparisons. As can be seen, the proposed features outperformed the state-of-the-art benchmark on all five tasks by a substantial margin, with the exception of the *N* vs. AD1 task where only a subtle improvement was obtained with the experiment 4 settings. Overall, these results suggest that making the mask generic (i.e., settings in experiments 1 and 4) can lead to improved accuracy on unseen data. This was the case for four out of the five tasks, with the exception being the *N* vs. AD2 task, where experiment 2 settings resulted in the best results. Ultimately, having very specialized maps (e.g., as in experiment 3 settings) did not lead to accuracy levels that could not be achieved with other less specialized settings.

### 3.6. Age-Related Confounds

Ultimately, it is important to gauge if the proposed features are indeed measuring neural changes due to AD and not solely due to normal aging. Such evaluation is particularly important in settings where age matching is not possible between groups, as is the case herein. Previous studies have shown a direct link between normal aging and changes in EEG powers and frequencies (e.g., see [58, 59]), thus we explore a linear mapping between the proposed feature and age and test if the obtained results correspond to those obtained from a random mapping function.  Table 8 shows the root mean square errors (between estimated and true age) obtained for the Experiment 1 and Experiment 4 settings. None of the tests showed significant differences from chance at a 99% confidence level, thus suggesting that the proposed features are not capturing normal aging-related changes in the EEG and are indeed capturing neurodegenerative insights. Similar findings were found for the other two experiment settings and are omitted here for the sake of brevity.

### 3.7. Study Limitations and Future Work

The results reported in this study were performed on a limited sample size of 54 participants. While the results are promising, they need to be validated on a larger dataset. Open-source EEG datasets for AD are not widely available; thus, future work should focus on creating open-source datasets. The recent international push to enable EEG as a clinical biomarker [16] may enable more widespread collection that will help push the research forward. Furthermore, as is widely known, the machine learning algorithms are sensitive to hyper-parameter tuning. Since the data available for this study was limited, not much effort was spent on parameter optimization; hence, the accuracy results reported may not be the highest achievable with the proposed feature set. Once more data become available, future work could explore the overall impact of hyperparameter tuning on classification accuracy. Moreover, in this study, saliency maps per channel were obtained, but an average map was used for simplified feature importance selection. Once more data becomes available, per-channel optimized feature patches may be used, resulting in improved accuracy. It is known that degeneration due to AD can spread to different brain regions as the disease progresses, hence it is expected that different regional saliency maps will be useful for the different tasks explored herein. This channel-aware optimization may also lead to an overall system that can rely on a subset of the EEG channels, hence, enabling the creation of a portable, low-density, and low-cost solution, as in [69], that could help tackle AD worldwide.

Moreover, here we explored AD1 vs. AD2 discrimination, hence, obtaining insights on how disease severity can change the EEG patterns used in biomarker development. Ultimately, future work should explore neural changes seen in longitudinal studies where data from one patient's progression is monitored, thus truly leading to disease progression insights. For example, it is known that roughly a quarter of patients with mild cognitive impairment (MCI) will progress to AD within a 4-year window. Understanding the neural signature changes between MCI patients that do progress to AD and those that do not could provide useful clues not only for disease progression but also for the risk associated with developing AD at very early stages. Lastly, recent studies have shown that EEG combined with MRI could lead to useful insights for disease severity level prediction [70]. On that study, the authors showed the complementarity of the visually obtained patch features with anatomical features extracted from MRIs to predict the MMSE scores of the patients. Future work should explore the benefits of combining the proposed features with MRI ones to quantify the gains that can be achieved.

## 4. Conclusions

In this article, we have proposed the use of a convolutional neural network (CNN) combined with saliency maps, trained on an image-like power modulation spectrogram of eyes-closed resting-state EEGs, to find new biomarkers of Alzheimer's disease. The goal was to explore if a data-driven biomarker selection method could provide insights complementary to those obtained via visual inspection. In particular, we explored biomarkers for five classification tasks: healthy (*N*) vs. mild-AD (AD1) vs. moderate-to-severe AD (AD2), (2) *N* vs. AD (combined AD1 and AD2), (3) *N* vs. AD1, (4) AD1 vs. AD2, and (5) *N* vs. AD2. The biomarkers found were extracted for each of the available EEG channels and reduced to the top 24 features via feature selection before being input to a support vector machine for final classification. The most important brain regions found coincided with those widely reported in the AD literature, and most importantly, the power modulation spectrogram patches complemented those found previously via visual inspection. Overall, the proposed method outperformed the benchmark on all five classification tasks and by a large margin. To assure the newly-proposed features were not measuring EEG changes due to normal aging, results were compared to a random age prediction classifier and no significant differences were found.

## Figures and Tables

**Algorithm 1:**
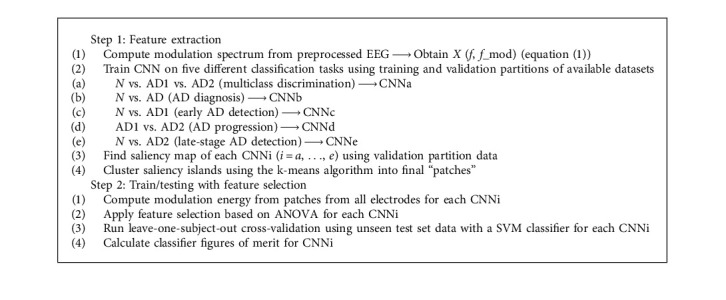
Summary of methodology steps.

**Table 1:** Participant demographic details.

Group identifiers	Subjects (female)	Age (years)	Education (years)	MMSE
*N*	20 (9)	68.0 ± 8.6	10.1 ± 5.5	28.5 ± 1.7
AD1	19 (11)	74.1 ± 5.5	5.6 ± 2.8	19.4 ± 5.3
AD2	15 (9)	75.0 ± 11.8	4.1 ± 3.8	12.8 ± 5.0

**Table 2:** CNN hyper-parameter tuning details.

Hyper-parameters	Range explored	Chosen
Kernel_size	(3.3), (5.5), (7.7), (9.9)	(3,3)
Regularizers.l2	(1*e* − 2), (1*e* − 4)	(1*e* − 2)
Dropout	(65%, 75%, 85%, 90%)	85%
Optimizer	Adam, Nadam, Adagrad, Adamax	Nadam
Learning rate	(0.01, 0.001, 0.0001)	0.0001
Batch_size	4, 8, 32, 64	4
Epochs	20, 30, 40, 50	50

**Table 3:** CNN accuracy on the validation set for the five tasks.

Tasks	Acc (%)	F1 (%)
T1	90.5	90.5
T2	87.3	84.6
T3	91.4	91.0
T4	92.5	91.4
T5	93.0	91.6

**Table 4:** Best combination of threshold (Th) and number of clusters (C) for each of the four experiments.

Tasks	*Exp. 1*		*Exp. 2*		*Exp. 3*		*Exp. 4*	
	Th (%)	C	Th (%)	C	Th (%)	C	Th (%)	C
T1	92	3	92	3	92	3	92	3
T2	96	4	96	4	96	5	96	5
T3	86	3	84	4	92	4	96	3
T4	94	5	86	5	82	4	86	4
T5	96	3	92	4	94	3	90	4

**Table 5:** Top 24 features selected using the experiment 1 settings.

Rankings	*N* vs. AD1 vs. AD2	*N* vs. AD	*N* vs. AD1	AD1 vs. AD2	*N* vs. AD2
1	R3-C4_1_	R2-F7_2_	R2-F7_3_	R2-Oz_4_	R3-C4_5_
2	R3-P3_1_	R3-F7_2_	R1oR2-F7_3_	R2-Fz_4_	R3-P3_5_
3	R3-Fz_1_	R2-C4_2_	R1oR3-Oz_3_	R2-F4_4_	R3-C3_5_
4	R3-Oz_1_	R1-C4_2_	R1oR3-Pz_3_	R2-P3_4_	R3-F3_5_
5	R3-F3_1_	R3-C4_2_	R1oR3-O2_3_	R3oR2-C3_4_	R3-P4_5_
6	R3-C3_1_	R1-F7_2_	R2-C4_3_	R2-F3_4_	R3-T4_5_
7	R3-F4_1_	R2-T4_2_	R1oR3-O1_3_	R4oR2-C3_4_	R2-C4_5_
8	R3-P4_1_	R4-C4_2_	R2-T3_3_	R2-C3_4_	R3-F4_5_
9	R3-Cz_1_	R2-F3_2_	R1oR2-T4_3_	R2-O2_4_	R2-F3_5_
10	R3-T4_1_	R2-T3_2_	R1oR2-C4_3_	R2-Pz_4_	R3-Oz_5_
11	R3-O1_1_	R3-T4_2_	R1oR2-C3_3_	R5oR2-C3_4_	R3-Fz_5_
12	R3-O2_1_	R3-F3_2_	R1oR2-F3_3_	R2-O1_4_	R2-P3_5_
13	R3-Pz_1_	R2-T6_2_	R2-T6_3_	R3oR1-C3_4_	R2-T4_5_
14	R2-C4_1_	R1-T4_2_	R3-F7_3_	R2-Cz_4_	R1-F3_5_
15	R1-F7_1_	R2oR3-F8_2_	R1oR2-T3_3_	R1oR2-C3_4_	R3-T5_5_
16	R2-F3_1_	R3-T3_2_	R2-T4_3_	R1oR2-F7_4_	R3-O1_5_
17	R2-fz_1_	R4-F7_2_	R1oR3-Fp1_3_	R5oR3-C3_4_	R1-C4_5_
18	R1-C4_1_	R2-C3_2_	R1oR2-T6_3_	R2-P4_4_	R2-Fz_5_
19	R1-F3_1_	R4-T4_2_	R1oR3-P4_3_	R3oR2-F7_4_	R3-T6_5_
20	R2-P3_1_	R2oR3-F7_2_	R3oR2-T3_3_	R4oR1-C3_4_	R2-C3_5_
21	R1-T4_1_	R1-F3_2_	R3oR2-F7_3_	R5oR4-C3_4_	R3-Cz_5_
22	R2-T4_1_	R3-T6_2_	R1oR2-Fp1_3_	R2-C4_4_	R1-T4_5_
23	R3-T5_1_	R2-P3_2_	R1oR2-T5_3_	R1-Oz_4_	R2-F4_5_
24	R2-F4_1_	R2oR3-T4_2_	R1oR2-F8_3_	R5oR3-T5_4_	R2-P4_5_

*Number of features per brain region—experiment 1 settings*					
Frontal	8	9	8	5	7
Central	5	5	3	11	6
Temporal	4	9	8	1	5
Parietal	4	1	2	3	4
Occipital	3	0	3	4	2

**Table 6:** Top 24 features selected using experiment 4 settings.

Rankings	*N* vs. AD1 vs. AD2	*N* vs. AD	*N* vs. AD1	AD1 vs. AD2	*N* vs. AD2
1	R2-Oz_1_	R4oR3-O1_2_	R1-Fp2_3_	R1-Fz_4_	R4-C3_5_
2	R2-Fz_1_	R4-F7_2_	R1-Pz_3_	R1-Oz_4_	R4-P3_5_
3	R2-P3_1_	R4oR3-Pz_2_	R1-Fz_3_	R1-F4_4_	R4-T5_5_
4	R2-P4_1_	R4oR2-C4_2_	R2oR3-Oz_3_	R1oR4-Fz_4_	R4-P4_5_
5	R2-C4_1_	R4oR3-Oz_2_	R1-O2_3_	R1-P3_4_	R4-O1_5_
6	R2-F4_1_	R4oR2-T6_2_	R1oR3-Pz_3_	R1-F3_4_	R4-C4_5_
7	R2-F3_1_	R4-C4_2_	R2oR3-O1_3_	R1-O2_4_	R4oR3-C3_5_
8	R2-O2_1_	R4oR2-T5_2_	R1-Cz_3_	R3-Oz_4_	R4-F3_5_
9	R2-Pz_1_	R4oR2-T3_2_	R2oR3-O2_3_	R3-Fz_4_	R4-Oz_5_
10	R2oR3-Fz_1_	R4-T4_2_	R2oR3-P4_3_	R1-Pz_4_	R4oR3-F3_5_
11	R2oR3-F3_1_	R2oR1-O1_2_	R1-Oz_3_	R1oR4-Oz_4_	R4-T4_5_
12	R2-C3_1_	R4oR2-O1_2_	R2oR3-Pz_3_	R1-Cz_4_	R4-T6_5_
13	R2-O1_1_	R4oR2-P4_2_	R1oR3-Oz_3_	R1-C3_4_	R4-F7_5_
14	R2-Cz_1_	R4oR2-T4_2_	R2-O2_3_	R1-O1_4_	R4-O2_5_
15	R2oR3-C3_1_	R4oR3-F4_2_	R1oR3-O2_3_	R1oR4-F3_4_	R4-Pz_5_
16	R2oR3-Oz_1_	R2oR1-O2_2_	R1oR3-Fp1_3_	R1oR4-F4_4_	R4-F4_5_
17	R2oR3-C4_1_	R5oR2-C4_2_	R1oR3-Fp2_3_	R1oR4-C3_4_	R4oR3-T4_5_
18	R2oR3-F4_1_	R4oR3-O2_2_	R2-T5_3_	R1-P4_4_	R4oR3-C4_5_
19	R2oR3-P3_1_	R4oR3-Fp1_2_	R1oR3-Fz_3_	R1oR4-P3_4_	R4oR3-O1_5_
20	R2-T4_1_	R4oR5-O1_2_	R2-O1_3_	R3oR4-Fz_4_	R4oR3-P3_5_
21	R1oR3-F3_1_	R4-T6_2_	R2-P4_3_	R1oR4-O1_4_	R4-T3_5_
22	R2oR3-cz_1_	R4oR2-P3_2_	R1oR3-T3_3_	R3oR2-C3_4_	R4-Fz_5_
23	R2oR3-O1_1_	R4oR3-P4_2_	R1oR2-T6_3_	R1-C4_4_	R4oR3-F4_5_
24	R1-C4_1_	R4-P3_2_	R2-Oz_3_	R1oR4-Pz_4_	R4-Cz_5_

*Number of features per brain region—experiment 4 settings*					
Frontal	7	3	5	8	6
Central	7	3	1	5	5
Temporal	1	6	3	0	5
Parietal	4	5	5	5	4
Occipital	5	7	10	6	4

**Table 7:** Performance comparison achieved with best threshold-cluster settings and top-24 features. Bold values indicate the highest accuracy achieved for a given classification task.

Tasks	Benchmark [29]		Proposed (exp. 1)		Proposed (exp. 2)		Proposed (exp. 3)		Proposed (exp. 4)	
	Acc (%)	F1 (%)	Acc (%)	F1 (%)	Acc (%)	F1 (%)	Acc (%)	F1 (%)	Acc (%)	F1 (%)
T1	50 ± 3	50 ± 3	**54** ± **2**	**55** ± **2**	**54** ± **2**	**55** ± **2**	**54** ± **2**	**55** ± **2**	47 ± 2	49 ± 2
T2	70 ± 1	65 ± 1	60 ± 2	49 ± 3	60 ± 2	49 ± 3	**71** ± **2**	**61** ± **2**	**71** ± **2**	**61** ± **2**
T3	64 ± 3	63 ± 3	48 ± 2	48 ± 2	46 ± 2	46 ± 2	46 ± 4	46 ± 4	**65** ± **2**	**64** ± **2**
T4	73 ± 2	71 ± 2	**83** ± **2**	**83** ± **2**	82 ± 0	82 ± 0	**83** ± **1**	**83** ± **1**	79 ± 1	79 ± 1
T5	73 ± 2	72 ± 2	86 ± 3	86 ± 3	**89** ± **3**	**89** ± **3**	85 ± 3	84 ± 3	82 ± 2	81 ± 2

Bold values indicate the highest accuracy achieved for a given classification task.

**Table 8:** Age prediction accuracy comparison against a random regression model for experiment 1 and experiment 4 settings.

Tasks	Exp. 1		Exp. 4	
	Nonrandom	Random	Nonrandom	Random
T1	10.33 ± 2.02	9.94 ± 1.55	9.82 ± 1.65	9.71 ± 1.75
T2	9.83 ± 1.85	9.74 ± 1.45	9.40 ± 1.69	9.48 ± 1.65
T3	8.05 ± 1.20	8.55 ± 1.55	7.81 ± 1.18	8.19 ± 1.36
T4	9.74 ± 2.77	9.30 ± 2.42	9.70 ± 2.64	9.26 ± 2.63
T5	12.19 ± 2.63	11.97 ± 2.14	11.28 ± 1.82	11.63 ± 2.46

## Data Availability

The data used to support the findings of this study are available upon request to Tiago.Falk@inrs.ca.
